# Anti-Inflammatory Therapy Modulates Nrf2-Keap1 in Kidney from Rats with Diabetes

**DOI:** 10.1155/2016/4693801

**Published:** 2016-02-03

**Authors:** Abraham Said Arellano-Buendía, Montserrat Tostado-González, Fernando Enrique García-Arroyo, Magdalena Cristóbal-García, María Lilia Loredo-Mendoza, Edilia Tapia, Laura-Gabriela Sánchez-Lozada, Horacio Osorio-Alonso

**Affiliations:** ^1^Laboratory of Renal Physiopathology, Juan Badiano 1, 14080 Mexico City, DF, Mexico; ^2^Department of Nephrology, Instituto Nacional de Cardiología-Ignacio Chávez, Juan Badiano 1, 14080 Mexico City, DF, Mexico; ^3^Histopathology Laboratory, Research Subdivision, School of Medicine, Universidad Panamericana, Donatello 43, 03910 Mexico City, DF, Mexico

## Abstract

This study addressed the relationship of proinflammatory cytokines and Nrf2-Keap1 system in diabetic nephropathy. The experimental groups were control, diabetic, and diabetic treated with mycophenolate mofetil (MMF). The renal function, proinflammatory and profibrotic cytokines, oxidative stress, morphology, and nephrin expression were assessed. Diabetic group showed impaired renal function in association with oxidative stress and decreased Nrf2 nuclear translocation. These results were associated with increased mesangial matrix index, interstitial fibrosis, and increased nephrin expression in cortex and urine excretion. Additionally, interleukin-1*β*, IL-6, and transforming growth factor-*β*1 were increased in plasma and kidney. MMF treatment conserved renal function, prevented renal structural alterations, and partially prevented the proinflammatory and profibrotic cytokines overexpression. Despite that MMF treatment induced nephrin overexpression in renal tissue, preventing its urinary loss. MMF salutary effects were associated with a partial prevention of oxidative stress, increased Nrf2 nuclear translocation, and conservation of antioxidant enzymes in renal tissue. In conclusion, our results confirm that inflammation is a key factor in the progression of diabetic nephropathy and suggest that treatment with MMF protects the kidney by an antioxidant mechanism, possibly regulated at least in part by the Nrf2/Keap1 system, in addition to its well-known anti-inflammatory effects.

## 1. Introduction

Diabetic nephropathy (DN) is the leading cause of end-stage renal failure in developed countries. Traditionally, interactions among metabolic and hemodynamic factors are considered to be involved in the development of renal lesions in patients with diabetes. However, several other factors such as oxidative stress and inflammatory processes have been shown to play important roles in the pathogenesis of DN; these factors are not completely independent but they interact with each other [1–3]. Several studies report the infiltration of macrophages and proinflammatory cells in kidney at different stages of DN [4–6]. The inflammatory infiltrate produces reactive oxygen species (ROS), proinflammatory cytokines, and growth factors, which lead to upregulation of chronic systemic inflammation and mediate the progression of diabetic nephropathy [4].

As a consequence of inflammation, a variety of cytokines and acute phase proteins are released in order to augment or attenuate the inflammatory response [7]. The main inflammatory cytokines involved in the development of DN are interleukin 1 (IL-1), IL-6, and IL-18 and tumor necrosis factor-*α* (TNF-*α*); these might contribute to the progression of renal injury either directly or indirectly [1, 8–10].

Thus, chronic inflammation contributes to DN not only as a consequence of a direct effect of proinflammatory mediators on cellular signaling but also by creating a state of oxidative stress.

In recent years, several investigators have provided substantial evidence implicating nuclear factor (erythroid-derived 2)-like 2 (Nrf2), a redox-sensitive transcription factor, in inflammation and associated disorders [11]. Nrf2 is a basic region-leucine zipper-type transcription factor belonging to the cap “n” collar (CNC) family that tightly interacts with Kelch-like ECH-associated protein 1 (Keap1) [12, 13]. When Keap1 is exposed to electrophiles, reactive oxygen species (ROS), reactive nitrogen species (RNS), or heavy metals, cysteine residues in Keap1 are modified, leading to conformational changes in the complex that, in turn, prevent proteasome-mediated degradation of Nrf2 [14]. Accordingly, Nrf2 is stabilized, allowing for accumulation in the nucleus and coordinate induction of a wide array of genes encoding antioxidant proteins, all of which can counteract inflammatory and oxidative damage by enhancing the removal of cytotoxic electrophiles or ROS in cells [15, 16].

The therapeutic potential of Nrf2 activation in diabetes, implicating control of oxidative stress, in addition to regulation of inflammatory cytokines as methods of Nrf2 protection, was described [2, 15, 17]. Also, it has been described that severe oxidative stress is associated with inflammation in chronic kidney disease (CKD).

Oxidative stress and inflammation are mediators in the development and progression of chronic kidney disease (CKD) and its complications, and they are inseparably linked as each begets and amplifies the other.

Although pathways involved in intrarenal ROS production and inflammation in experimental CKD have been widely explored, the relationship of DN on proinflammatory cytokines and Nrf2-Keap1 system in diabetes is poorly studied. Therefore, the present study was undertaken to address this issue.

## 2. Methods

### 2.1. Reagents

Streptozotocin (STZ), 4-nitrophenyl-N-acetyl-*β*-D-glucosaminide, 4-hydroxynonenal (4-HNE), and 2,4-dinitrophenylhydrazine (DNPH) were purchased from Sigma (St. Louis, MO, USA). All other chemicals used were of the highest analytical grade available.

### 2.2. Experimental Design

All animal procedures were performed in accordance with the Mexican Federal Regulation for Animal Experimentation and Care (NOM-062-ZOO-2001) and were approved by the Bioethics and Investigation Committees of the Instituto Nacional de Cardiología “Ignacio Chávez.”

Adult male Wistar rats were used at 10–14 weeks of age (250–300 g). Animals were randomly distributed into four groups (*n* = 16 each group): control (C), diabetes (D), diabetes treated with mycophenolate mofetil (DMMF) (15 mg/kg body weight, daily by gastric gavage), and diabetes treated with insulin (DI). Diabetes was induced by a single administration of STZ (50 mg/kg i.p.) dissolved in citrate buffer (0.1 M, pH 4.5). The control group received the same volume of citrate buffer. The MMF (CellCept®) is insoluble in water by which it was suspended in water by vigorous agitation immediately before use. Insulin (Humulin; Eli Lilly and Company, Indianapolis, IN, USA) was administered i.p. in an initial dose of 6 international units (IU) followed by 2 to 4 IU daily, depending on morning blood glucose values.

Blood glucose concentration was determined using Medisense Optium Xceed (Abbott Alameda, CA, USA) 72 h after STZ administration and only rats with glucose measurements over 20.0 mmol/L were considered with diabetes for further studies.

Treatment was initiated after confirmation of diabetes. To analyze early renal changes induced by diabetes, a group of animals were sacrificed after 1 week (7 days) of diabetes confirmation (8 rats/group). The remaining rats were studied after 1 month of diabetes confirmation (8 rats/group). All experimental groups were maintained on laboratory diet and water* ad libitum*.

Rats were placed in metabolic cages (Nalgene, Rochester, NY, USA) and urine (24 h) was collected at 1 week and 1 month after diabetes induction. Urine samples were centrifuged at 5,000 g for 15 minutes to remove debris, and the supernatant was analyzed. The urinary variables measured were diuresis, glycosuria (IL 300 plus, Clinical Chemistry Analyzer, Holliston, MA, USA), albuminuria (Albumin Rat ELISA Kit, Abcam, Cambridge, MA, USA), and the urinary excretion of N-acetyl *β*-D-glucosaminidase (NAG).

### 2.3. Measurement of NAG Activity

For the determination of NAG activity in urine samples, 4-nitrophenyl-N-acetyl-*β*-D-glucosaminide was used as substrate. One unit of enzymatic activity (U) represents the amount of enzyme, which hydrolyses one *μ*mol of substrate per min at 37°C [18]. The results were expressed as U/24 h.

### 2.4. Glomerular Filtration Rate (GFR)

The GFR was estimated by polyfructosan clearance method as previously described [18].

### 2.5. Evaluation of IL-1*β*, IL-6, and TGF-*β*1

Total proteins from plasma or renal tissue were determined using the Bradford method. Equal amounts of protein (7.5 *µ*g) were denatured in gel loading buffer by heating at 85°C for 5 minutes before loading onto 10% sodium dodecyl sulfate SDS-polyacrylamide gels. After electrophoretic separation, samples were transferred to polyvinylidene difluoride (PVDF) membranes and incubated at 4°C overnight with primary antibody (1 : 1,000) diluted in phosphate buffered saline with Tween® 20. The antibodies used were IL-1*β*, IL-6, and TGF-*β*1. The protein bands were visualized with enhanced chemiluminescence reagents (ECL Plus Western Blotting Detection System, GE Healthcare Bio-Sciences Piscataway, NJ, USA), and analysis and intensity quantification were conducted using Kodak Electrophoresis Documentation and Analysis System 290 (EDAS 290). Housekeeping protein *β*-actin was used as a loading control. Positive immunoreactive bands were quantified densitometrically and expressed as the ratio of problem to *β*-actin in arbitrary units.

### 2.6. Evaluation of Renal Markers of Oxidative Stress

#### 2.6.1. Determination of Lipid Peroxidation by Measuring 4-Hydroxynonenal (4-HNE) Levels

For the 4-HNE assay 50 mg of kidney cortex or medulla was homogenized in ice-cold phosphate buffered saline (PBS) and the colorimetric assay was performed as previously described [18]. The results were expressed as nmol of 4-HNE/mg protein.

#### 2.6.2. Assay of Oxidized Protein by Measuring Carbonyl Protein Content

The determination of carbonyl groups in the proteins was measured using the reaction with 2,4-dinitrophenylhydrazine (DNPH) as previously described [18]. Protein carbonyl groups were estimated by using the molar absorption coefficient of 22,000 M^−1^·cm^−1^ for DNPH derivatives, and its concentration was expressed as nmol carbonyl groups/mg protein. Guanidine solution was used as a blank.

### 2.7. Semiquantitative Assay of Nrf2-Keap1 System and Nephrin

#### 2.7.1. Preparation of Renal Tissue

Cortex was washed thoroughly with ice-cold saline, 10% (w/v), and later was homogenized in a Potter-Elvehjem homogenizer in ice-cold 50 mM phosphate buffer pH 7.4 containing mammalian protease inhibitor cocktail. The homogenates were used for the determination of total protein concentration by the Bradford method and the antibodies used were anti-Nrf2, anti-Keap1, anti-Heme Oxygenase-1 (HO-1), anti-catalase (CAT), anti-Cu-Zn-superoxide dismutase (SOD-1), anti-glutathione peroxidase (GPx-1), and anti-nephrin. Housekeeping protein *β*-actin was used as a loading control. Positive immunoreactive bands were quantified densitometrically and expressed as the ratio of problem to *β*-actin in arbitrary units. Urinary excretion of nephrin was assayed by immunoblotting as previously described [18]. To account for differences in hydration and urine concentration, the results were normalized to urine creatinine.

The frozen kidney was ground to a powder and then mixed in ice-cold HEPES buffer (10 mM HEPES, 0.2% Triton X-100, 50 mM NaCl, 0.5 mM sucrose, 0.1 mM EDTA, protease, and phosphatase inhibitors) and homogenized with an ice-chilled Dounce homogenizer at 4°C. An aliquot of the homogenate was stored and the rest was used to make cytosolic and nuclear extracts. This was spun at 10,000 rpm for 10 min, and the supernatant was aliquoted and stored at −70°C as the cytosolic extract. The pellet was suspended in ice-cold buffer (10 mM HEPES, 500 mM NaCl, 10% glycerol, 0.1 mM EDTA, 0.1 mM EGTA, 0.1% IGEPAL, and protease and phosphatase inhibitors) and vortexed at 4°C for 15 min and centrifuged for 10 min at 14,000 rpm. The resulting supernatant was aliquoted and stored as the nuclear extract at −70°C. A small aliquot of kidney homogenate, cytosolic, and nuclear extract was kept at 4°C for protein estimation. Nuclear extracts were used for Nrf2 immunoblotting. Intensity values were normalized to Histone 3 (H3) and expressed as arbitrary units of protein expression.

#### 2.7.2. Histological Assessment of Renal Injury

The kidneys from experimental groups were fixed in 10% formalin in PBS and embedded in paraffin. Sections of 3 *µ*m thick were obtained and stained with periodic acid-Schiff (PAS) and Sirius red. To evaluate the glomerular and mesangial areas, photomicrographs of PAS stained sections were taken at a 200x magnification, from nonoverlapping fields. Thirty glomeruli were assessed in each rat kidney. Glomerular size and mesangial areas were, respectively, determined, by manually tracing the tuft perimeter and automatically measuring the PAS positive material in each glomerulus with a computerized image analysis software (Axiovision Rel. 4.8.2, Zeiss). The mesangial matrix index represented the ratio of mesangial matrix area divided by the tuft area. For interstitial fibrosis assessment, 10 cortex fields images (200x magnification) at random were recorded from Sirius red stained sections of each kidney. Interstitial fibrosis consisted in red stained areas (collagen I and collagen III) [19] located between tubules and its quantification was performed using the same software mentioned above. Proportion of fibrosis was calculated by dividing the area of interstitial fibrosis by the total area per field, excluding the glomerular and luminal tubular areas. Microscopic images were obtained using a digital camera mounted on a light microscope (Axiophot 2, Zeiss, Germany). All slides were analyzed in a blinded fashion.

#### 2.7.3. Immunofluorescence

Immunofluorescence analysis of Nrf2 in the renal tissue was performed using 3 *μ*m sections from paraffin embedded renal tissue. After deparaffinization and rehydration the samples were submitted to heat induced antigen retrieval in sodium citrate buffer (10 mM, pH 6.0) during 20 minutes. Next steps were incubation during 1 hour with a blocking solution (2% bovine serum albumin, 5% normal goat serum), 1 hour with the primary antibody (anti-Nrf2 rabbit polyclonal GeneTex GTX 103322) at a 1/50 dilution, and 1 hour with a secondary FITC-conjugated goat anti-rabbit antibody at a 1/100 dilution; all these reagents were diluted in TBS with 0.05% Tween 20. Then the Tyramide Signal Amplification fluorescence kit from Perkin Elmer (NEL741E001KT) was used according to manufacturer instructions. Briefly an anti-fluorescein HRP-conjugated antibody was applied to the sections (30 min), followed by incubation for 3 minutes with the amplification reagent. All the steps were performed at room temperature and the slides were washed with TBS with 0.05% Tween 20 after each step, except after the application of the blocking solution. To nuclear counterstaining DAPI was used. The same microscope and software mentioned above for the histological assessment were utilized for the fluorescence labeled sections analysis.

### 2.8. Statistical Analysis

Data are expressed as the mean ± SEM. Statistical differences among groups were calculated using ANOVA with Bonferroni correction (Prism 5.0; GraphPad Software, San Diego, CA, USA). Significance for all statistical comparisons was set at *p* < 0.05.

## 3. Results

### 3.1. Metabolic Parameters

At short term (1 week) there was a significant increase (^*∗*^ *p* < 0.05) in the mean plasma glucose level, diuresis, and glycosuria in the group with diabetes compared to the control group (Table 1). On the other hand, body weight and creatinine in the group with diabetes showed no significant difference compared to control, MMF-treated, and insulin-treated group (data not shown).

One month later, the group with diabetes had low body weight and increased blood glucose levels, diuresis, and glycosuria in comparison with control (Table 1, ^*∗*^ *p* < 0.05).

There was no statistically significant difference in body weight, blood glucose, and diuresis between the group with diabetes untreated and MMF-treated group (Table 1).

### 3.2. Effects of Diabetes on Renal Function

At short term (1 week) creatinine levels were unchanged in the diabetic group which suggests that short-term diabetes was not associated with renal dysfunction (Table 1); therefore GFR, albuminuria, and NAG urinary excretion were not measured.

After one month of diabetes onset, the total GFR (polyfructosan clearance), albuminuria, and urinary NAG excretion (Table 1) were increased in the group with diabetes. The MMF treatment showed a renoprotective effect by a lower increase in total GFR, albuminuria, and NAG urinary excretion, compared with untreated group (Table 1). However, these values did not reach those of the control group.

### 3.3. Effect of Diabetes on Proinflammatory and Profibrotic Cytokines

**Figure 1:**
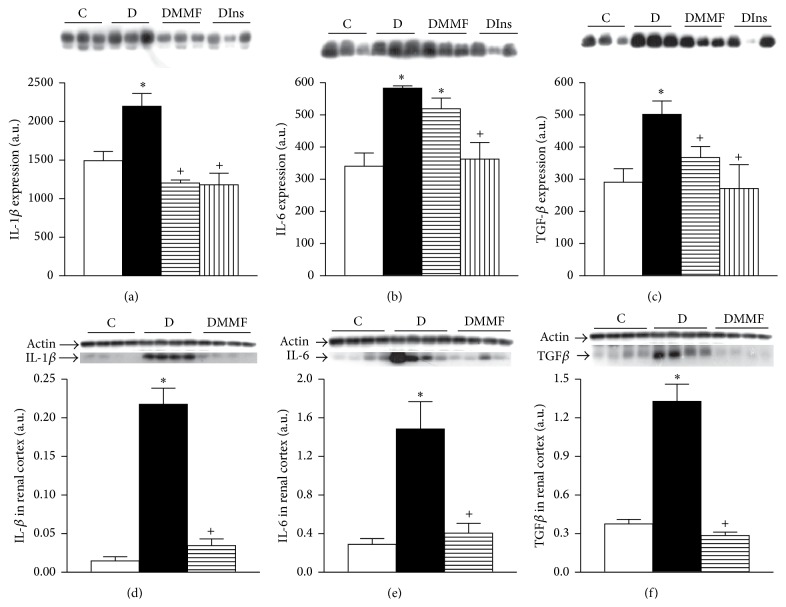
Analysis of the proinflammatory and profibrotic cytokines. Representative immunoblot and quantitative analysis of protein expression of interleukin 1*β* (a, d), interleukin 6 (b, e), and transforming growth factor-*β* (c, f) in plasma (a, b, c) and renal cortex (d, e, f), respectively. C: control, D: diabetic, DMMF: diabetic MMF-treated, and DIns: diabetic insulin-treated. Data are expressed as mean ± SEM, *n* = 8;  ^*∗*^ *p* < 0.05 versus C; ^+^ *p* < 0.05 versus D.

Diabetes did not induce changes neither in inflammatory cytokines (IL-1*β* and IL-6) nor in the profibrotic cytokine TGF-*β*1 at short term (1 week) (data not shown). However, one month later western blotting analysis showed an increase in the amount of immunoreactive peptide for IL-1*β*, IL-6, and TGF-*β*1 in plasma of groups with diabetes (Figures 1(a), 1(b), and 1(c), resp.). The MMF treatment reduced IL-1*β* and TGF-*β*1 protein expression (Figures 1(a), 1(b), and 1(c), resp.) compared with group with diabetes untreated. The insulin treatment prevented all diabetes induced alterations (Figures 1(a), 1(b), and 1(c), resp.).

The effect of diabetes on proinflammatory and profibrotic cytokines in renal tissue is shown in Figures 1(d), 1(e), and 1(f). Western blotting analysis demonstrated a significant increase in the amount of immunoreactive peptide corresponding to gene expression of IL-1*β*, IL-6, and TGF-*β*1 in renal tissue from group with diabetes compared to the control group (Figures 1(d), 1(e), and 1(f), resp.). The MMF treatment caused a significant lowering of renal proinflammatory and profibrotic cytokines compared to the diabetes state alone (Figures 1(d), 1(e), and 1(f), resp.).

### 3.4. Renal Histopathology

Mesangial matrix expansion and interstitial fibrosis are two of the hallmarks of diabetic nephropathy and were therefore investigated in group with diabetes in the present study via analysis of kidney sections.

**Figure 2:**
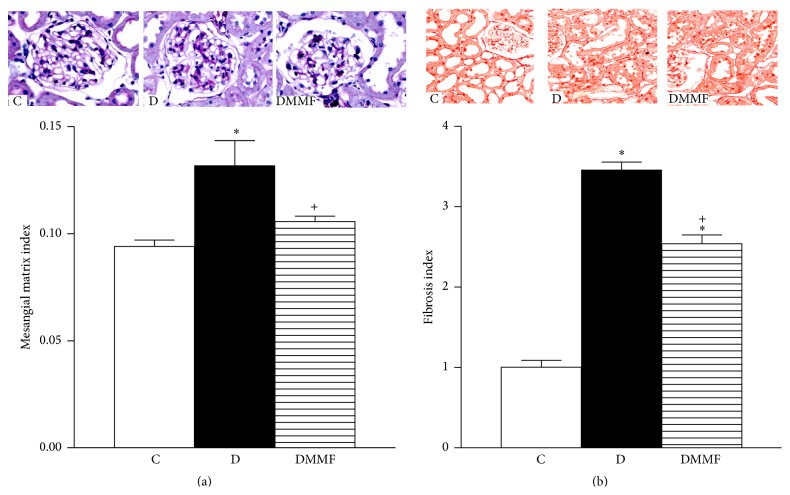
Histopathological analysis. Mesangial matrix index, images of histopathological glomerular changes, and quantitative analysis (a). Kidney sections show mild but noticeable increase in mesangial PAS positive area in diabetes (D) in comparison with control (C). Treatment with MMF demonstrates a reduction in the mesangium (DMMF). Interstitial fibrosis, kidney cortical sections, and quantitative analysis (b). Images show very thin red lines surrounding tubular epithelium (interstitium) in control (C), an increase in the space occupied by interstitium in diabetes (D), and finally a mild decrease in tubular interstitium in diabetes MMF-treated (DMMF). Original magnifications 200x. ^*∗*^ *p* < 0.001 versus C; ^+^ *p* < 0.001 versus D.

Our glomerular morphometric study demonstrated that the glomerular area was not significantly different in the three experimental groups (data not shown) but that the mesangial matrix index was significantly elevated in group with diabetes in comparison to that of the control group (one month) (^*∗*^ *p* < 0.001). The increase of mesangial matrix index was suppressed in the group with diabetes treated with MMF compared with the untreated group (^+^ *p* < 0.001) (Figure 2(a)). Tubular microscopic changes (not quantified) were characterized by widened lumen, epithelial vacuolar degeneration, and cellular detachment.

Fibrosis assessment showed that as early as one month of diabetes induction, the interstitial fibrosis index was significantly increased in the cortex of the group with diabetes in comparison to that of the control group (^*∗*^ *p* < 0.001). There was less interstitial fibrosis in the group with diabetes MMF-treated compared with the untreated group (^+^ *p* < 0.001) (Figure 2(b)).

### 3.5. Evaluation of Oxidative Stress and the Expression of Nrf2-Keap1 System in Kidney

4-HNE and carbonyl proteins are biochemical indicators of lipid and protein oxidation, respectively, and these are markers of oxidative stress.

In the short-term studies (1 week), no statistically significant changes in oxidative stress markers in the renal cortex and medulla were observed between all experimental groups (Table 1).

However, after one month of diabetes onset, the accumulation of lipid peroxides and protein oxidation content was increased in renal cortex and medulla from group with diabetes compared to the control group (Table 1). The MMF treatment significantly attenuated the accumulation of lipid and protein oxidized in comparison with the group with diabetes untreated (Table 1).

**Figure 3:**
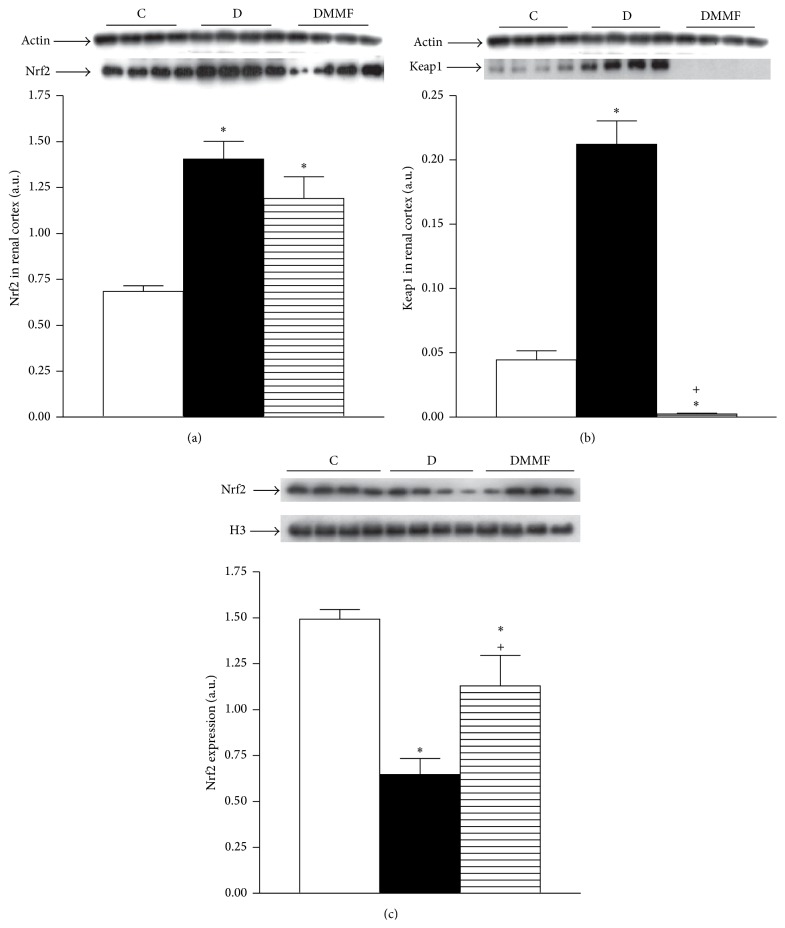
Renal expression of Nrf2 and Keap1. (a) Cytoplasmic Nrf2 expression, (b) cytoplasmic Keap1 expression, and (c) Nrf2 expression. Representative immunoblot and quantitative analysis of relative protein expression. C: control, D: diabetic, and DMMF: diabetic MMF-treated. Data are expressed as mean ± SEM, *n* = 8;  ^*∗*^ *p* < 0.05 versus C; ^+^ *p* < 0.05 versus D.

We next analyzed the expression of Nrf2 and Keap1 in renal cortex, as this pathway is the master regulator of the antioxidant cellular protection. After one month of diabetes induction, in cortex from group with diabetes, the Nrf2 and Keap1 protein expression were higher than that of the control group (Figures 3(a) and 3(b)). The MMF treatment did not change Nrf2 protein levels (Figures 3(a) and 3(b)), but it significantly decreased the Keap1 protein expression compared with group with diabetes untreated and control group (Figures 3(a) and 3(b)).

In the nuclei-enriched fraction, the Nrf2 protein expression was decreased in diabetic rats, compared with the control group. The MMF treatment increased the Nrf2 expression when compared with the diabetic untreated (Figure 3(c)).

Additionally, we compared the renal Nrf2 expression and localization between diabetic and diabetic MMF-treated groups by immunofluorescence.

**Figure 4:**
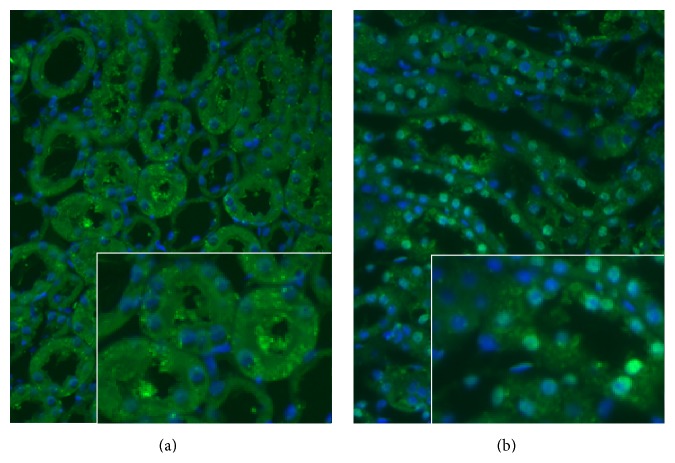
Representative immunofluorescence photomicrographs for Nrf2 in renal cortex show the presence of FITC (green) reactivity in the untreated diabetic group tubular epithelial cells cytoplasm, mainly in the proximal tubule brush border (a), and the change in its localization to the nucleus in the diabetic MMF-treated group tubular cells (b). Nuclear counterstaining with DAPI in blue. Original magnification 200x/1.6 zoom.

The immunofluorescence labeling results showed that in the group with diabetes the Nrf2 expression is mainly in the cytoplasmic and very low in nuclei from tubular epithelial cells. In contrast, in the diabetic MMF-treated group the main reactivity is nuclear (Figure 4).

**Figure 5:**
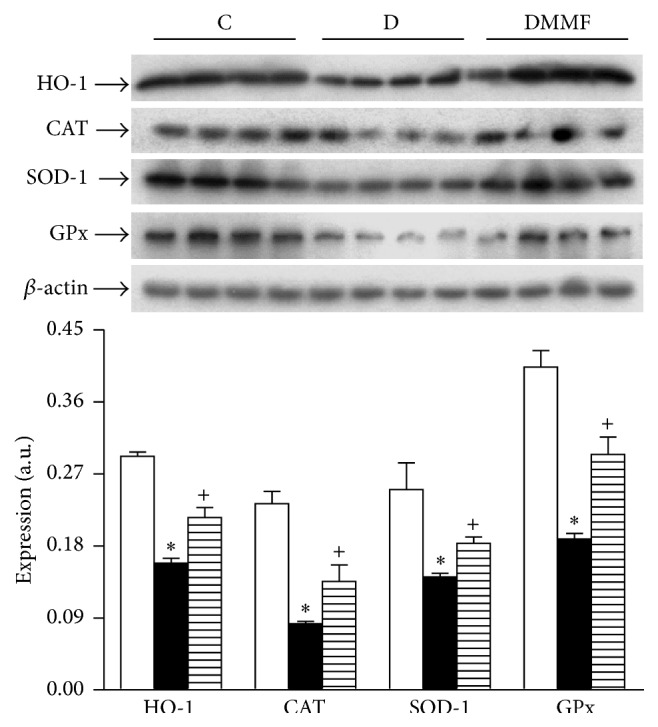
Analysis of cytoprotective enzymes Nrf2-regulated in renal cortex. Representative immunoblot and quantitative analysis of relative protein expression. C: control, D: diabetic, and DMMF: diabetic MMF-treated. Data are expressed as mean ± SEM, *n* = 8; ^*∗*^ *p* < 0.05 versus C; ^+^ *p* < 0.05 versus D.

In addition, to investigate whether the transcription of Nrf2-target genes was impaired in diabetic rats, the expression of cytoprotective antioxidant enzymes was assessed. The protein expression of HO-1, CAT, SOD-1, and GPx-1 was decreased in diabetic rats compared with control group. The MMF treatment resulted in significantly higher expression of HO-1, CAT, SOD-1, and GPx-1 compared with diabetic untreated animals (Figure 5).

### 3.6. Evaluation of Nephrin in Diabetes

At one month of diabetes onset, the nephrin expression was increased in renal cortex compared with control group (Figure 4). This result was associated with a higher urinary excretion of nephrin in group with diabetes compared with control group (Figure 4). The MMF treatment increased nephrin expression in cortex and prevented the urinary loss of nephrin in group with diabetes compared with untreated group (Figure 4).

## 4. Discussion

Oxidative stress and inflammation are mediators in the development and progression of chronic kidney disease (CKD) and its complications, and they are inseparably linked as each begets and amplifies the other. Increases in oxidative stress can induce the production of inflammatory cytokines and likewise an increase in inflammatory cytokines can stimulate the production of free radicals. Thus, the present study was undertaken to address the relationship of inflammation and oxidative stress on progression of diabetic nephropathy.

**Figure 6:**
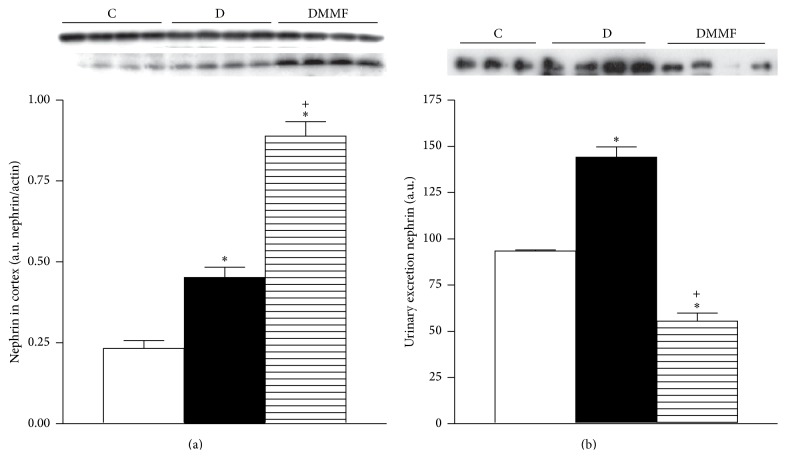
Analysis of nephrin: (a) renal cortex and (b) urinary excretion. Representative immunoblot and quantitative analysis of relative protein expression. C: control, D: diabetic, and DMMF: diabetic MMF-treated. Data are expressed as mean ± SEM, *n* = 8;  ^*∗*^ *p* < 0.05 versus C; ^+^ *p* < 0.05 versus D.

In this study, the increase in proinflammatory and profibrotic cytokines in plasma and tissue was associated with impaired renal function as well as increase in urinary excretion of nephrin, mesangial matrix index, and interstitial fibrosis (see Figure 6). Furthermore, lipids and protein oxidation were increased in association with a failure of Nrf2 transcription factor to translocate to nuclei, despite a significant increase of cytoplasmic Nrf2 expression in cortex from group with diabetes. Treatment with anti-inflammatory MMF attenuated the effects diabetes induced at systemic and renal levels and partially prevented hyperfiltration, albuminuria, and the urinary NAG excretion. We also found a significant association between the reduction of albuminuria and the urinary excretion of nephrin, as well as in expression of TGF-*β*1 and cytokines. The anti-inflammatory treatment decreased the oxidative stress, mesangial matrix index, and interstitial fibrosis. However, it did not affect Nrf2 expression, but a marked decrement in the Keap1 protein expression was induced. Moreover, the MMF treatment increased the Nrf2 accumulation and therefore stimulated the expression of antioxidant enzymes such as HO-1 CAT, SOD, and GPx-1.

Thus, we conclude that the nephroprotective effects induced by MMF treatment could be mediated at least partially by the effect of treatment on the Nrf2 pathway and the negative regulation of TGF-*β*1, IL-1*β*, and IL-6 possibly by the nuclear factor-*κ*B (NF-*κ*B) pathway.

Besides contributing to the knowledge of the anti-inflammatory effects of MMF in the progression of diabetic nephropathy, we also suggest an additional mechanism by which MMF is capable of providing nephroprotective effects, in addition to the well-described mechanisms [5, 6, 20–22].

Oxidative stress associated with diabetes is secondary to increased ROS production and diminished antioxidant capacity [18]. The latter is largely caused by impaired Nrf2 activation and translocation, the transcription factor that regulates genes encoding antioxidant and detoxifying molecules [2, 3, 23]. However, we observed an increase in Nrf2 and Keap1, its repressor, which could explain the increase in oxidative stress, despite increase in Nrf2. Similar data has been described in studies* in vitro*, where after Nrf2 activation by antioxidant, an increase in Keap1 expression was detected [24]. Upregulation of Nrf2 in response to hyperglycemia was found not only in cultured cells but also in the kidney of mice with diabetes [25]. Other studies have shown slightly increased Nrf2 expression in mice with diabetes at 2 weeks and 2 months after diabetes onset [26, 27]. On the other hand we observed lower Nrf2 expression in the nuclear fraction and kidney slices from diabetic rats likely due to an impaired signaling for Nrf2 translocation to nuclei. These results suggest that in diabetic state Nrf2 is adaptively trying to remain functional to overcome diabetic damage at the early stage of diabetes. However, its repressor Keap1 is also upregulated. Not surprisingly, the expression levels of an array of key antioxidant enzymes under the control of Nrf2 were also reduced (HO-1 CAT, SOD-1, and GPx-1), suggesting that oxidative stress is partially caused by altered Nrf2 expression and nuclear translocation.

On the other hand, the oxygen derivatives may create a vicious circle acting as second messengers, because it has been shown that hydrogen peroxide induces the IL-6 promoter by activating NF-*κ*B through nuclear factor-*κ*B inducing kinase and activator protein-1 (AP-1) leading to the transcription of genes encoding cytokines, growth factors, and extracellular matrix proteins (ECM) [28, 29]. The inflammation could cause structural damage through the release of proinflammatory and profibrotic cytokines as well as ROS such as hydrogen peroxide, superoxide, and hydroxyl radicals, all of which may contribute to renal injury.

Given the role of oxidative stress and inflammation in pathogenesis and progression of DN, interventions aimed at restoring these pathological mechanisms may be effective in retarding end-stage renal disease progression.

In this regard, MMF treatment showed beneficial effects mediated by its anti-inflammatory properties; nevertheless, MMF effects on Nrf2-Keap1 pathway also contributed to its salutary outcomes. The MMF treatment did not affect Nrf2 expression; however, it induced a marked decrement in Keap1 expression. Therefore we hypothesize that MMF induces the Nrf2 stabilization and its subsequent translocation into the nucleus; however, further analysis is needed to elucidate this point. Accumulated Nrf2 binds to a cis-acting antioxidant/electrophile responsive element and activates the transcription of a cluster of genes encoding for antioxidant and cytoprotective enzymes, all of which can counteract inflammatory and oxidative damage by enhancing the removal of cytotoxic electrophiles or ROS in cells [15, 16]. Accordingly we found an increased expression of HO-1 CAT, SOD-1, and GPx-1 in MMF-treated diabetic rats.

On the other hand, there are at least three mechanisms that could explain the downregulation of Keap1 induced by MMF: (1) ubiquitination of Keap1 in parallel with inhibition of Keap1 dependent ubiquitination of Nrf2 [30], (2) increased binding of sequestosome 1 to Keap1 promoting its ubiquitination [31], and finally (3) as has been demonstrated, phosphorylation and dephosphorylation of tyrosine 141 causing destabilization and degradation of Keap1, leading to the release of Nrf2 [32]. However, the effect of MMF on the degradation pathway and turnover profile of Keap1 remains to be elucidated. Modulation of Nrf2 signaling may have profound effects on other redox-sensitive inflammation-regulating transcription factors, such as NF-*κ*B and AP-1 [15, 33].

Podocyte slit diaphragm-associated proteins play a central role in maintaining the size-selective barrier, and podocyte injury or activation contributes to albuminuria and overt proteinuria in DN. Our results show an increase in nephrin in renal cortex as well as urine in diabetic group, suggesting that slit-pore composition is perturbed by hyperglycemia, inflammation, oxidative stress, and hyperfiltration. Following a change in slit-pore composition, this destabilizes and consequently disrupts the glomerular filtration barrier structure, thereby leading to podocyte loss, nephrinuria, and albuminuria. We hypothesize that, as a protective mechanism, the nephrin expression is upregulated to counteract the loss and maintain the glomerular filtration barrier functional. The MMF treatment enhances the nephrin expression in cortex and prevents its urinary loss. Other studies report that MMF treatment reduces glomerular expression of desmin/MCP-1, increases the expression of nephrin and podocin, prevents the loss of podocytes, attenuates mesangial expansion and profibrotic mRNA expressions, and subsequently reduces urinary albumin excretion [5, 6, 21, 22].

An interesting finding is that the proinflammatory and profibrotic cytokines in plasma and kidney were decreased by the anti-inflammatory treatment and correlate with the renal function and oxidative stress. Additionally, we show the effect of anti-inflammatory treatment (MMF) on Nrf2 a master regulator of cellular detoxification responses and redox status which has been suggested that may induce anti-inflammatory effects in DN.

## 5. Conclusions

Our results demonstrate that MMF treatment was able to ameliorate glomerular injury and interstitial fibrosis and minimize inflammation and oxidative stress possibly in Nrf2-Keap1 dependent fashion. Furthermore, our results describe an additional mechanism for anti-inflammatory therapy protection through the Nrf2 pathway and negative regulation of TGF-*β*1, IL-1*β*, and IL-6.

The beneficial role of anti-inflammatory treatment in protecting against the loss of nephrin is thought to arise from its antioxidant and anti-inflammatory effects on podocytes. Furthermore, even though previous studies showed a podocyte-protective effect of MMF administration, the underlying mechanism has not been revealed.

## Figures and Tables

**Table 1:** General and biochemical parameters in the experimental groups at short- and long-term diabetes induction.

	C		D		DMMF	
	7 days	30 days	7 days	30 days	7 days	30 days
Body weight (g)	282 ± 17.82	428 ± 18.18	294.9 ± 3.46	273.5 ± 7.28^*∗*^	288.8 ± 12.67	295.8 ± 9.53^+^

Blood glucose (mg/dL)	85.7 ± 12.37	90.58 ± 14.27	420 ± 35^*∗*^	438 ± 28.27^*∗*^	390.0 ± 15.73^+^	368.9 ± 17.85

Serum creatinine (mg/dL)	0.40 ± 0.08	0.45 ± 0.02	0.45 ± 0.01	0.43 ± 0.06	0.42 ± 0.02	0.48 ± 0.05

Diuresis (mL/24 hrs)	16.66 ± 2.4	17.8 ± 6.8	23.86 ± 2.29	39.68 ± 5.05^*∗*^	21.37 ± 1.2	27.95 ± 4.32

Glucosuria (mg/dL)	0 ± 0	0 ± 0	4817 ± 180.9^*∗*^	4791 ± 189.8	4302.50 ± 164.5	4377 ± 169.5

Total GFR (mL/min)	ND	1.31 ± 0.063	ND	3.51 ± 0.46^*∗*^	ND	2.51 ± 0.12^*∗*+^

Albuminuria (mg/24 h)	ND	163.6 ± 18.89	ND	508.7 ± 48.44^*∗*^	ND	294.3 ± 21.20^*∗*+^

Urinary NAG (U/24 h)	ND	0.098 ± 0.031	ND	0.758 ± 0.08^*∗*^	ND	0.432 ± 0.05^*∗*+^

Oxidized proteins in ctx (DNPH nmol/mg protein)	2.57 ± 0.69	0.10 ± 0.01	3.75 ± 0.70	974.3 ± 53^*∗*^	3.27 ± 0.52	745.9 ± 51.19^+^

Oxidized proteins in med (DNPH nmol/mg protein)	11.37 ± 3.8	18.78 ± 5.51	9.17 ± 3.4	179.8 ± 24.95^*∗*^	8.62 ± 2.9	166.4 ± 13.65^*∗*+^

Lipid peroxidation in ctx (4-HNE nmol/mg protein)	0.42 ± 0.18	0.22 ± 0.1	0.90 ± 0.28	5.51 ± 0.23^*∗*^	0.73 ± 0.3	3.39 ± 0.24^*∗*+^

Lipid peroxidation in med (4-HNE nmol/mg protein)	0.48 ± 0.22	0.022 ± 0.005	1.0 ± 0.18	3.44 ± 0.55^*∗*^	1.34 ± 0.38	1.91 ± 0.08^*∗*+^

C: control group, D: group with diabetes, DMMF: group with diabetes treated with mycophenolate mofetil, GFR: glomerular filtration rate, NAG: N-acetyl *β*-D-glucosaminidase, ND: not determined, ctx: renal cortex, med: renal medulla, DNPH: dinitrophenylhydrazone, and 4-HNE: 4-hydroxynonenal. Data are mean SEM of 8 animals in each group. ^*∗*^ *p* < 0.05 versus control; ^+^ *p* < 0.05 versus D.
